# Maternal separation affects expression of stress response genes and increases vulnerability to ethanol consumption

**DOI:** 10.1002/brb3.841

**Published:** 2017-11-30

**Authors:** Taciani de Almeida Magalhães, Diego Correia, Luana Martins de Carvalho, Samara Damasceno, Ana Lúcia Brunialti Godard

**Affiliations:** ^1^ Laboratório de Genética Animal e Humana Departamento de Biologia Geral Universidade Federal de Minas Gerais Belo Horizonte MG Brazil

**Keywords:** ethanol, gene expression, HPA axis, maternal separation, postnatal stress, reward system

## Abstract

**Introduction:**

Maternal separation is an early life stress event associated with behavioral alterations and ethanol consumption. We aimed to expand the current understanding on the molecular mechanisms mediating the impact of postnatal stress on ethanol consumption.

**Methods:**

In the first experiment (T1), some of the pups were separated from their mothers for 6 hr daily (Maternal Separation group ‐ MS), whereas the other pups remained in the cage with their respective mothers (Control group ‐ C). In the second experiment (T2), mice from both groups were subjected to the model of free‐choice between water and sucrose solution or between water and ethanol solution. Maternal behavior was assessed at the end of T1. At the end of both T1 and T2, pups were subjected to the light/dark box behavioral test and blood corticosterone concentrations were analyzed.

**Results:**

Our maternal separation protocol led to intense maternal care and affected weight gain of the animals. The expression of stress response genes was altered with higher levels of *Crh* and *Pomc* being observed in the hypothalamus, and higher levels of *Crhr1*,* Crhr2*,* Htr2a* and lower levels of *Nr3c1* and *Htr1a* being observed in the hippocampus after T1. At the end of T2, we observed higher levels of *Avp* and *Pomc* in the hypothalamus, and higher levels of *Crhr1*,* Crhr2*,* Nr3c1*,* Slc6a4, Bdnf* and lower levels of *Htr1a* in the hippocampus. Additionally, maternal separation increased vulnerability to ethanol consumption during adolescence and induced changes in anxiety/stress‐related behavior after T2. Furthermore, voluntary ethanol consumption attenuated stress response and modified expression of reward system genes: enhancing *Drd1* and *Drd2*, and reducing *Gabbr2* in the striatum.

**Conclusion:**

Maternal separation induced behavioral changes and alterations in the expression of key genes involved in HPA axis and in the serotonergic and reward systems that are likely to increase vulnerability to ethanol consumption in adolescence. We demonstrated, for the first time, that ethanol consumption masked stress response by reducing the activity of the HPA axis and the serotonergic system, therefore, suggesting that adolescent mice from the MS group probably consumed ethanol for stress relieving purposes.

## 1 INTRODUCTION

Early life stress events, such as parental negligence, can active the hypothalamic‐pituitary‐adrenal axis (HPA) and change the regulation of genes with important roles for the HPA axis function and, therefore, can have a profound impact on the development and maturation of the central nervous system (CNS) (Frodl & O'Keane, 2013; Maccari, Krugers, Morley‐Fletcher, Szyf, & Brunton, 2014).

When activated by a stressful condition, the HPA axis stimulates secretion of corticotrophin‐releasing hormone (CRH) and vasopressin (AVP) by the hypothalamic paraventricular nucleus (Tomas, Newton, & Watson, 2013). CRH activates the surface receptors CRHR1 and CRHR2 which, along with AVP, is essential for the secretion of adrenocorticotropic hormone (ACTH) by the anterior pituitary (Tsigos & Chrousos, 2002). ACTH is produced by the enzymatic cleavage of pro‐opiomelanocortin (POMC) and is transported to the adrenal cortex where it stimulates the secretion of glucocorticoids: cortisol in humans and corticosterone in rodents (Tomas et al., 2013). Corticosterone binds to the receptor NR3C1 in the hippocampus and inhibits the release of CRH and POMC through an inhibitory feedback loop (Brunton, 2013).

The HPA axis can also be indirectly activated by serotonergic projections (Herman, Ostrander, Mueller, & Figueiredo, 2005). In these projections, serotonin (5HT) interacts with its pre‐ (5HT1A) and postsynaptic (5HT2A) receptors and with the serotonin transporter (5HTT), thereby modulating stress response and the activation of the HPA axis (Lesch, Araragi, Waider, van den Hove, & Gutknecht, 2012). Activation of these serotonergic receptors may also mediate regulation of brain‐derived neurotrophic factor (BDNF) and it has been suggested that the regulation of neurotrophins can also contribute to the effects of stress (Cavus & Duman, 2003).

In addition, exposure to stress hormones during postnatal period is thought to be associated with behavioral alterations, such as increased vulnerability to ethanol consumption in adolescence and adult life (Nylander & Roman, 2013). Ethanol consumption can change transcriptional regulation in the mesolimbic brain regions causing euphoric effects (positive reinforcements) and stress relieving effects (negative reinforcements) (Bendre, Comasco, Nylander, & Nilsson, 2015). These are mediated by dopaminergic neurons, which originate in the ventral tegmental area (VTA) and project to the nucleus accumbens (NAcc), increasing dopaminergic transmission and altering GABAergic terminals via GABA receptors (GABAA and GABAB) (Koob & Volkow, 2010; Nestler, 2005; Spanagel, 2009).

Given the importance of postnatal stress as a predictor of ethanol consumption in adolescence and adult life, several studies using rodent models of maternal separation have tried to elucidate how postnatal stress can influence the expression of genes involved in ingestion and anxiety‐related behaviors and stress response (Daniels et al., 2009; Deussing & Wurst, 2005; Maccari et al., 2014).

Previous studies in rodents have shown the effects of postnatal stress and ethanol consumption on the HPA axis and on gene expression in the dopaminergic system (Bendre et al., 2015; García‐Gutiérrez et al., 2015; Todkar et al., 2015). Nevertheless, these studies were limited as they analyzed only a small set of genes and did not correlate the results with serotonergic transmission genes or possible behavioral changes. Furthermore, to understand how genes involved in stress response might contribute to the vulnerability to ethanol consumption, it is necessary evaluate their expression and regulation following voluntary ethanol intake. So far, studies of maternal separation and vulnerability to ethanol consumption have been inconsistent and have not provided a comprehensive assessment of how postnatal stress can increase susceptibility to ethanol consumption in adolescence (Harrison & Baune, 2014; Tractenberg et al., 2016). Therefore, here we performed behavioral analyses and evaluated the expression of genes of the serotonergic system and the HPA axis following maternal separation and exposure to ethanol in an attempt to understand the molecular mechanisms mediating the impact of postnatal stress on ethanol consumption. We hypothesized that postnatal stress could affect the regulation of the HPA axis and the serotonergic system leading to increased ethanol consumption, which, in turn, would affect the reward system, thus, intensifying the seeking behavior and further increasing the individual's vulnerability to ethanol consumption.

We used an adapted version of the maternal separation protocol (Nylander & Roman, 2013), in this sense we kept the control group in the same cage as the MS group is thought to mimic conditions in the wild (Tractenberg et al., 2016). During the period of MS, half the litter half was kept in their cages with their mothers and the other half was submitted to maternal separation, being placed in a new cage, without the mothers, between the days post (PNDs) 5 and 21 with duration of 6 hr daily. We evaluated: (i) the effects of maternal separation on maternal and offspring behavior; (ii) offspring corticosterone levels; (iii) changes in ethanol consumption associated with maternal separation stress; and (iv) examined, for the first time, the effects of ethanol consumption on stress response (Firk & Markus, 2007; Greetfeld et al., 2009; Murgatroyd et al., 2009; Wu, Patchev, Daniel, Almeida, & Spengler, 2014) and the reward system genes, and investigated the associated biochemical and behavioral alterations.

## 2 MATERIALS AND METHODS

### 2.1 Animals

Nine C57BL/6 females (six to eight weeks old) provided by the Animal Facility of the Federal University of Minas Gerais (UFMG) were used to generate the 42 pups analyzed in this study. Females were kept in individual cages along with males from the same strain, and checked daily for pregnancy. Animals were kept under a 12 hr’ light cycle (6 am to 6 pm) with food and water *ad libitum*.

**Figure 1:**
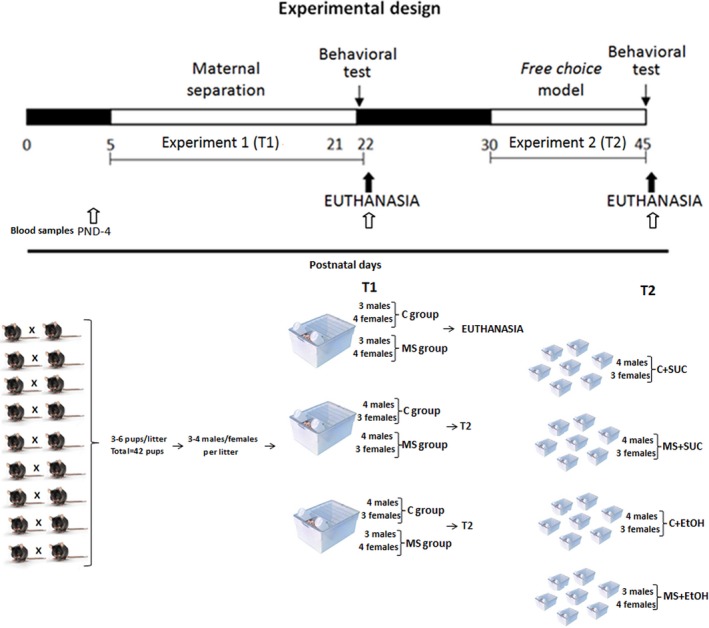
Diagram summarizing the experimental design. White areas represent the experimental periods (T1 and T2) defined by the postnatal days (PND). The first experiment (T1) encompassed the maternal separation protocol whereby the mothers were separated from their offspring for 6 hours daily (group MS). Animals from the control group (C) remained with their mothers. Experiment 2 (T2) consisted of the free‐choice model, in which the mice had to choose between water and 0.5% sucrose solution (C+SUC and MS+SUC) or between water and 10% ethanol solution (C+EtOH and MS+EtOH). At the end of T1 and T2 blood corticosterone levels were determined and the mice subjected to the light/dark box behavioral test. All experimental groups were composed of seven animals (*n *= 7)

In total, nine crosses were made to obtain 42 pups, 21 pups of the control group and 21 pups of the maternal separation group. As they were born, the pups were distributed (on postnatal day PND‐2) among three new cages (the experimental cages), each cage containing two dams. Therefore, each experimental cage (50 × 60 × 22 cm) held 14 pups and two dams. The pups were divided into two groups: the maternal separation group (MS, *n *= 7), and the control group (C, *n *= 7). At the end of the first experiment (T1, see below), seven pups from group MS and seven from group C were euthanized for analysis. The remaining 28 pups were, then, placed in individual cages and submitted to the free‐choice protocol. At the end of experiment T2 (PND‐45), the animals were euthanized (Figure 1).

The study was approved by the Ethics Committee of the UFMG (CEUA‐UFMG), protocol number 116/2015, and all measures were taken to minimize animals’ pain or discomfort.

### 2.2 Experimental design

The experimental design consisted of two experiments. In the first (T1), pups were submitted to an adapted protocol of maternal separation between PND‐5 and PND‐21 (Nylander & Roman, 2013). The 42 pups were divided into three experimental cages (*n *= 14), each containing two experimental groups: Control (C, *n *= 7) and Maternal Separation (MS, *n *= 7). All experimental groups were composed of at least three animals of each sex (*n *≥ 3). At the end of T1, 14 pups (seven from group MS and seven from group C) were euthanized for molecular analyses and the remaining 28 animals were placed in individual cages and submitted to the second experiment (T2), which comprised the model of free‐choice (Crabbe, Spence, Brown, & Metten, 2011) between water and ethanol solution or water and sucrose solution. Experiment T2 lasted from PND‐30 to PND‐45. Figure 1 summarizes the experimental design.

During the maternal separation protocol (T1), half of the litter (*n *= 7 in each experimental cage) were kept in their home cages with their mothers (C group), while the other half (*n *= 7 in each experimental cage) were subjected to MS, being placed in a new cage, without their mothers. The maternal separation protocol lasted for six hours each day, between 8am and 2 pm. Pups from group C were removed from their cages only for cage cleaning and weighting of the animals on PNDs‐5, 8, 11, 14, 17, and 20. On PND‐22, maternal care behavior was accessed, and pups were subjected to the light/dark box behavioral test after the MS protocol. Following the test, 14 pups (seven from de C group and seven from de MS group) were euthanized for molecular analyses.

In the second experiment (T2), the remaining 28 pups were placed in individual cages for one week (PND‐22 to PND‐29) for acclimatation. Animals were then subdivided into four groups (*n *= 7 pups per group), two of which (C+EtOH and MS+EtOH) had free‐choice between water and a 10% ethanol solution, while the other two (C+SUC and MS+SUC) had free‐choice between water and a 0.5% sucrose solution. The free‐choice protocol was applied 24 hr per day (Crabbe et al., 2011) and lasted for two weeks (between PND‐30 and PND‐45). We used sucrose solution as a control to evaluate the specific consumption of ethanol (rather than other palatable substances) following maternal separation (Walker et al., 2015). Fluid consumption was quantified daily by weighing the bottles. The positions of the bottles were changed daily in all groups to avoid position‐based favoritism by the pups. At the end of T2 (PND‐45), animals were subjected to the light/dark box behavioral test immediately after the end of the free‐choice protocol, and euthanized for further analyses.

### 2.3 Maternal behavior assessment

We assessed five maternal behaviors: (I) nesting; (II) self‐maintenance (grooming, eating, drinking and resting); (III) nursing (licking the pups and placing them on the nests); (VI) breastfeeding and (V) absence of contact with offspring. We evaluated the behavior of both females from each experimental cage (*n *= 6). Assessment was performed during the light cycle on days PND‐5, 14 and 20 (Stern, 1996).

We assessed maternal behavior before and after the maternal separation protocol for group MS, and before/after cleaning of the cages for group C (Stern, 1996). Each behavior was recorded as being present or not during the period of observation: preseparation (behavior present for at least 30 s during a period of observation of 15 min) and postseparation (behavior present for at least 15 s during a period of observation of 10 min). More than one behavior could be present during the same observation period, and they were all recorded.

### 2.4 Light/Dark box behavioral test

At the end of each experiment, we performed the light/dark box behavioral test, as described by Costall, Jones, Kelly, Naylor, and Tomkins (1989). Briefly, a box [46 × 27 × 30 cm (*l x b x h*)] was divided into two compartments: an “aversive area”, which represented two‐thirds of the total size, painted in white and illuminated by a 60W light bulb, and a smaller compartment painted in black and covered with a black lid. A 5‐cm opening separated the two compartments, allowing free transit between the light and dark areas. Mice were placed individually in the dark compartment facing the opening and the following variables were recorded for five minutes: latency (how long it took the mouse to go to the light compartment for the first time); the total amount of time spent in the light compartment; and the total number of transitions between compartments.

#### 2.4.1 Blood corticosterone concentration

Blood samples were collected through the submandibular plexus before the maternal separation scheme (PND‐4: baseline used as a control of hormone dosage), and on days PND‐22 and PND‐45. To avoid the influence of circadian fluctuations, all collections were made between 12 noon and 3 pm. Blood corticosterone concentration was determined using 25 μl of serum diluted 1:200 and the Corticosterone ELISA kit (Biogen, São Paulo, Brazil), following manufacturer's instructions.

#### 2.4.2 Brain dissection

At the end of T1 (PND‐22), brains from the euthanized animals were dissected on ice and the hypothalamus and hippocampus collected. After T2 (PND‐45) the brain regions collected were the hypothalamus, hippocampus, and the striatum, where the nucleus accumbens (Nacc) is located. All samples were stored at −80°C.

#### 2.4.3 Primer design

The sequences of the exons of the genes of interest were obtained from Ensembl Genome Browser (www.ensembl.org/), and primer pairs were designed using the program Primer3 v0.4.0 (Untergasser et al., 2012). The quality and specificity of the primer pairs were evaluated using the bioinformatics programs NetPrimer (www.premierbiosoft.com/netprimer/) and Primer‐BLAST (www.ncbi.nlm.nih.gov/tools/primer-blast/), respectively. Oligos were synthesized by IDT ‐ Integrated DNA Technologies (Síntese Biotecnologia, Belo Horizonte, Brazil). Primer sequences are shown in Table 1. Primers used for the GABA_B_ receptor subunit genes *Gabbr1* and *Gabbr2* were as defined by Ribeiro et al. (2012). The primers used for the reference genes (*Gapdh*,* Actb,* and *Ppia*) were designed and tested as previously described (Bibancos, Jardim, Aneas, & Chiavegatto,^2007^).

**Table 1:** Sequences of primers used in the qPCR

Genes	Forward sequence (5′–3′)	Reverse sequence (5′–3′)
*Gapdh (ID:95640)*	AGGAGCGAGACCCCACTAAC	GTGGTTCACACCCATCACAA
*Actb (ID:87904)*	GTGGGAATGGGTCAGAAGG	GGTCATCTTTTCACGGTTGG
*Ppia (ID:97749)*	AATGCTGGACCAAACACAAA	CCTTCTTTCACCTTCCCAAA
*Crh (ID:88496)*	GGCATCCTGAGAGAAGTCCC	GTTAGGGGCGCTCTCTTCTC
*Avp (ID:88121)*	TGCTACTTCCAGAACTGCCCA	TCCGAAGCAGCGTCCTTTG
*Pomc (ID:97742)*	GGGCTATGAACTTCGCAGG	GCAGAGTTTGGGAGGTGGTC
*Crhr1 (ID:88498)*	CGCAAGTGGATGTTCGTCTG	CTCCAGGACGTTTGCCAAA
*Crhr2 (ID:894312)*	CAAGTACAACACGACCCGG	AATGGGTTCGCAGTGTGAGT
*Nr3c1 (ID:95824)*	ACCTCACCACGGAGAGCAAC	GGGTTTTCAGTCAGGGGCT
*Htr1a (ID:96273)*	TTATCGCCCTGGATGTGCT	GCGTCCTCTTGTTCACGTAGTC
*Htr2a (ID:109521)*	ACCATAGCCGCTTCAACTCC	CGAATCATCCTGTAGCCCGA
*Slc6a4 (ID:96285)*	ATGGTTTGTGCTCATCGTGGT	CATACGCCCCTCCTGATGTC
*Bdnf (ID:88145)*	AGTGGAGCCGAACAAACTGATT	CGTTTACTTCTTTCATGGGCG

The IDs of the genes were obtained from Ensembl Genome Browser (www.ensembl.org/).

### 2.5 Molecular analyses

Total RNA was extracted from the brain samples using TRizol^®^, according to manufacturer's instructions (Invitrogen, São Paulo, Brazil). Samples were quantified using DeNovix DS‐11 (DeNovix, Delaware, EUA). For cDNA synthesis, 600 ng of total RNA were used in reverse transcription reaction with oligo (dT20) primers (Prodimol Biotecnologia, Belo Horizonte, Brazil) and Revertaid^®^ transcriptase (Thermo, São Paulo, Brazil), according to manufacturer's instructions.

Real‐time quantitative PCR (qPCR) was performed using Kapa SYBR^®^ Fastq PCR Kit Master Mix (Kapa Biosystems, São Paulo, Brazil) and the CFX 96TM Real Time system (BioRad). Thermo cycling parameters were as follows: 95°C for 3 min, followed by 40 cycles at 95°C for 3 s and 60°C for 20 s. Fluorescence levels were measured during the final step of each cycle (60°C). In all reactions, a negative control without cDNA template was tested. To normalize mRNA levels, the three reference genes glyceraldehyde‐3‐phosphate dehydrogenase (*Gapdh*), beta‐actin (*Actb*), and peptidylprolylisomerase A (*Ppia*) were used, and the relative quantity of the genes of interest was calculated as described by Vandesompele et al. (2002).

### 2.6 Statistical analyses

Obtained datasets were tested against the normal distribution using the Kolmogorov–Smirnov test. The weight (in grams) and ethanol consumption (in milliliters) were used to calculate the amount of ethanol consumed in grams per kilogram (g/kg). A two‐way ANOVA followed by Bonferroni's Correction Post hoc Test was used to analyze the weight gain and ethanol/sucrose consumption. The levels of corticosterone were analyzed using one‐way ANOVA followed by Bonferroni's Correction Post hoc Test. The maternal behavior, the light/dark box behavioral test, the preference for sucrose versus ethanol, and the mRNA transcript levels were analyzed using unpaired Student's t test. To complement these results, we performed two‐way ANOVA followed by Bonferroni's Correction Post hoc Test to evaluate the interactive effects of ethanol and postnatal stress in the light/dark box behavioral test and in the mRNA transcript levels (Graphics not shown). The treatment of outliers was done by calculating z scores. The data were represented as mean and standard error, and at 95% confidence interval (*p *< .05) was considered.

### 2.7 Methodological considerations

The maternal separation protocol adopted in our study was taken from that previously suggested by Nylander & Roman (2013).

The pups that we used as controls remained in the home cage with their mothers instead of being separated for short periods of 15 min (MS15). This option was used as a control in only 5.2% of the previous studies (Tractenberg et al., 2016), and only few authors opted for brief periods of separation as a control condition for MS. They argue that brief periods of separation during the postnatal period has a protective effect by inducing maternal care behavior towards the offspring and eliciting acute neuroendocrine activation (Kawakami, Quadros, Takahashi, & Suchecki, 2007; Meaney et al., 1991). Nevertheless, Todkar et al. (2015) reported more pronounced effects of maternal separation on stress‐related genes and ethanol consumption when they used a control litter that was not subjected to maternal separation, instead of the MS15 control. This confirms the previously reported effects of handling (Jaworski, Francis, Brommer, Morgan, & Kuhar, 2005; Pryce & Feldon, 2003) and highlights the importance of carefully choosing the control group. We, therefore, chose to keep control animals with their mothers (no maternal separation), and to maintain the animals from both groups C and MS in the same cage to reduce variability in maternal care and in the handling and cleaning procedures (Benner, Endo, Endo, Kakeyama, & Tohyama, 2014; Caldji, Diorio, & Meaney, 2000; Kawakami et al., 2007).

Another consideration is regarding the period during which the maternal separation occurs. The classic rodent model entails early separation of the pups from dams for two weeks (PND 1‐2 to PND 12‐14) (Nylander & Roman, 2013). However, the postnatal period is characterized by a stress hypo‐responsive period (SHRP), during which stressors are unable to induce increased secretion of corticosterone (Levine, 2001). In mice, the SHRP period occurs between PND‐1 and PND‐12 and, therefore, in the classical model, mice are hypo‐responsive during most of the maternal separation protocol. This may underlie the mouse resilience to maternal separation observed in previous studies (Own, Iqbal, & Patel, 2013; Own & Patel, 2013). Additionally, Tractenberg et al. (2016) reported that many studies of behavioral and biological phenotypes failed to show deleterious effects of maternal separation due to SHRP or differential maternal care. Indeed, shifting the maternal separation period to days PND‐4 to PND‐22 effectively increases stress response and alters HPA axis gene expression (MacQueen, Ramakrishnan, Ratnasingan, Chen, & Young, 2003). For these reasons, we performed maternal separation between the days PND‐5 and PND‐21, in an attempt to reduce the overlap with the SHRP period and to overcome resilience.

Another important aspect of our study was the assessment of maternal behavior, since variations in parental care following maternal separation can influence the neurobiological and behavioral outcomes in offspring (Caldji et al., 2000). Nevertheless, future studies are still needed to investigate whether maternal separation influences hormone and gene expression profiles of the mothers.

Considering that the vulnerability to ethanol consumption triggered by postnatal stress can be inappropriately deduced from a general vulnerability to rewards, which might include ethanol but also other sweetened substances (Porrino, Whitlow, & Samson, 1998; Ryan, Krstew, Sarwar, Gundlach, & Lawrence, 2014), we chose to adopt sucrose as a control to evaluate taste perception and motivation to consume palatable substance (Walker et al., 2015).

## 3 RESULTS

No statistically significant differences were observed in our data when animals were analyzed separated by gender (Figures S1, S2, S3, S4, and S5), thus indicating that the results were not influenced by sex. Therefore, we conducted the analyses with data from all the animals, regardless of their sex.

### 3.1 Effects of maternal separation and ethanol consumption in weight gain

**Figure 2:**
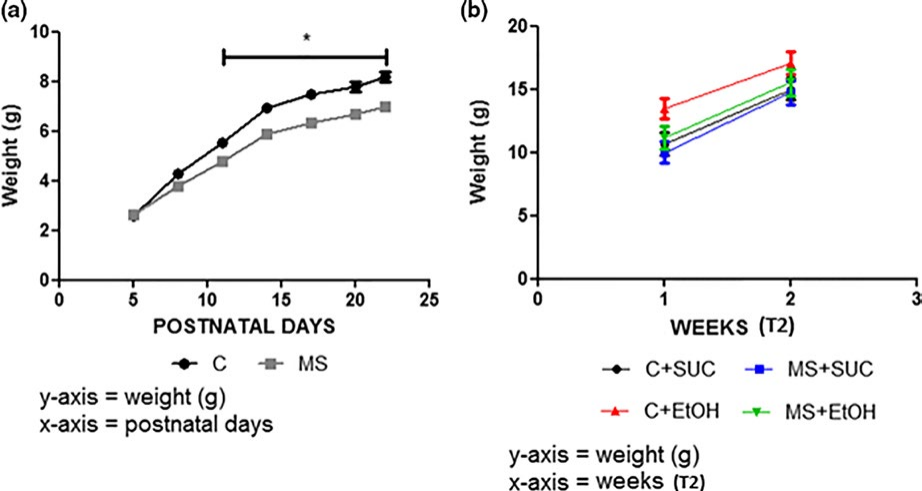
Analysis of weight gain (g) at the end of experiment 1 (T1) and experiment 2 (T2). (a) Weight gain (g) for groups MS and control (C) during T1. (b) Weight gain (g) for groups C+SUC, MS+SUC, C+EtOH and MS+EtOH during two weeks of T2. Experimental design is described in Figure 1. All experimental groups were composed of seven animals (*n *= 7). Data are expressed as mean and standard error. Two‐way ANOVA and Bonferroni's post hoc test correction were used to determine statistically significant differences between the groups in (a) and (b). *p < .05 C versus MS (T1)

We evaluated weight gain throughout the experiments (Figure 2) to determine if the stress of maternal separation and the consumption of ethanol or sucrose affected pups’ weight gain. During T1, pups from the group MS presented smaller weight gain (5.295 ± 0.610 g) compared with the pups from group C (6.111 ± 0.780 g) (*F*_6,69_ = 10.58, *p *< .05) (Figure 2a). No differences were observed in weight gain during T2 when comparing the groups C+SUC (12.85 ± 2.158 g), MS+SUC (12.37 ± 2.354 g), C+EtOH (15.28 ± 1.813 g) and MS+EtOH (13.36 ± 2.170 g) (*F*_3,45_ = 0.49 *p *> .05) (Figure 2b).

### 3.2 Effects of maternal separation in maternal behavior

**Figure 3:**
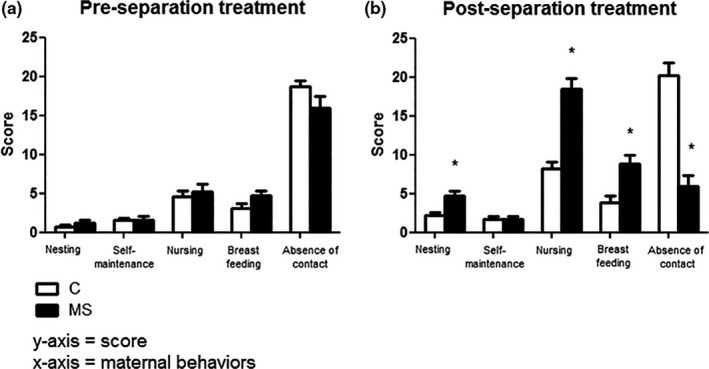
Maternal care behavior analysis before (a) and after (b) maternal separation (Group MS) and cage sanitization (Group C). Experimental design is described in Figure 1. Tests were conducted with two mothers per cage (*n *= 6 females). Data are expressed as mean and standard error. Student's t‐test was used to determine statistically significant differences between the groups in (a) and (b).*p < .05 C vs MS (T1)

We evaluated the maternal behavior of females from both the groups C and MS to determine if maternal response could alleviate the stress of separation. The average scores of the three days of documentation (PND‐5, PND‐14, and PND‐20), before and after the maternal separation protocol, are represented in Figure 3.

Before maternal separation, no differences were observed in any of the patterns of behavior analyzed between groups C (nesting: 0.666 ± 0.284; self‐maintenance: 1.583 ± 0.259; nursing: 4.583 ± 0.763; breastfeeding: 3.083 ± 0.679 and absence of contact with offspring: 18.67 ± 0.828) and MS (nesting: 1.167 ± 0.457, t = 0.92, df = 22, *p *> .05; self‐maintenance: 1.583 ± 0.543, t = 0.00. df = 22, *p *> .05; nursing: 5.250 ± 1.016, t = 1.59, df = 22, *p *> .05; breastfeeding: 4.667 ± 0.721, t = 0.52, df = 22, *p *> .05 and absence of contact with offspring: 16.17 ± 1.660, t = 1.34, df = 22, *p *> .05) (Figure 3a). However, maternal patterns of behavior were more intense and repetitive toward the pups that were separated from their mothers (Group MS ‐ nesting: 4.667 ± 0.655; nursing: 18.50 ± 1.384 and breastfeeding: 8.833 ± 1.120) compared with those who were kept with them the entire time (Group C ‐ nesting: 2.167 ± 0.441, t = 3.16, df = 22, *p *< .05; nursing: 8.167 ± 0.928, t = 3.47, df = 22, *p *< .05, and breastfeeding: 3.833 ± 0.928, t = 6.20, df = 22, *p *< .05) (Figure 3b). In addition, there was a significant decrease in the “absence of contact with offspring” in the MS group (6.0 ± 1.360) when compared with group C (20.25 ± 1.643, t = 6.68, df = 22, *p *< .05). Regarding the mothers’ self‐maintenance behavior (grooming, eating, drinking, and resting), no difference was observed between the groups (Group C – 1.833 ± 0.405 and group MS – 1.667 ± 0.414, t = 0.28, df = 22, *p *> .05).

### 3.3 Effects of maternal separation and ethanol consumption in Light/Dark Box Behavioral Test

**Figure 4:**
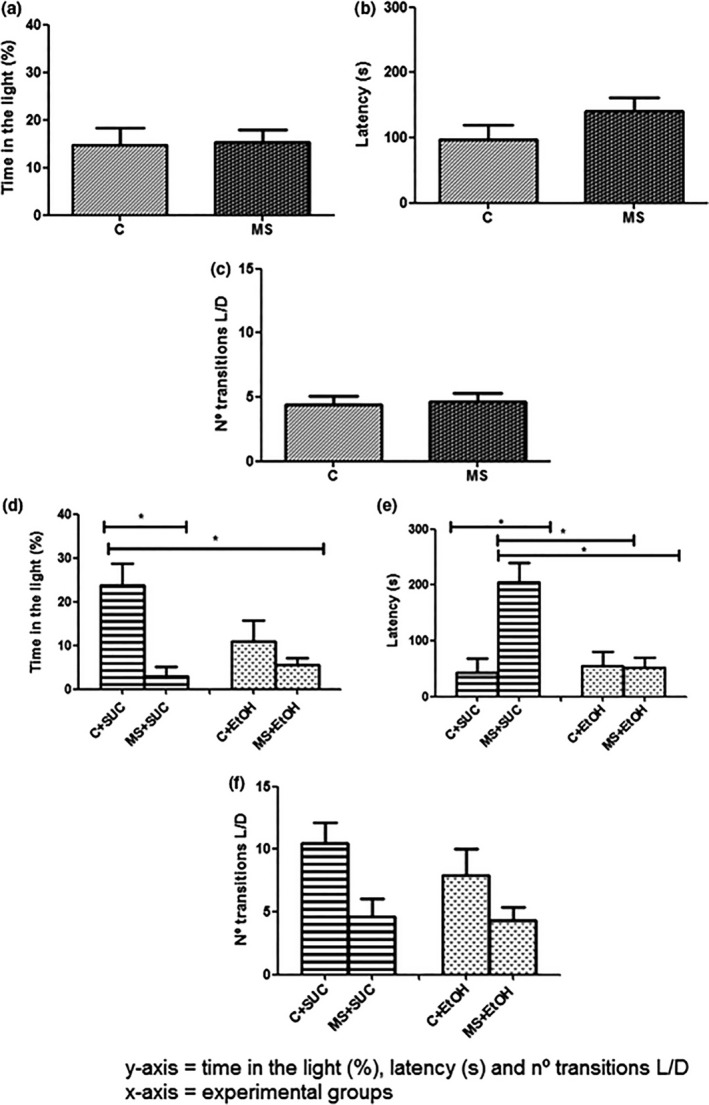
Light/dark box behavioral test at the end of experiment 1 (T1) and experiment 2 (T2). Proportion of time (%) spent in the light compartment following T1 (a) and T2 (d); Latency at the end of T1 (b) and T2 (e); and total number of transitions between the light and dark compartments following T1 (c) and T2 (f). Experimental design is described in Figure 1. All experimental groups were composed of seven animals (*n *= 7). Data are expressed as mean and standard error. Student's t‐test was used to analyze the differences between the groups C and MS in (a), (b) and (c) and between the groups C+SUC and MS+SUC and the groups C+EtOH and MS+EtOH in (d), (e) and (f). *p < .05 C+ SUC versus MS+ SUC (T2)

The light/dark box behavioral test was used to assess anxiety/stress‐related behavior changes following the maternal separation protocol (T1) and the model of free‐choice (T2) (Figure 4). At the end of T1, no differences were observed between the groups regarding the time spent in the light compartment (Group C – 14.79 ± 3.612% and Group MS – 15.38 ± 2.525%, t = 0.13, df = 37, *p *> .05), the latency (Group C – 97.65s ± 21.69 and Group MS – 140.90s ± 21.40, t = 1.41, df = 38, *p *> .05) and the number of compartment transitions (Group C – 4.36 ± 0.676 and Group MS – 4.60 ± 0.712, t = 0.23, df = 37, *p *> .05) (Figure 4a–c).

After T2, however, a significant difference was detected between the groups MS+SUC and C+SUC, with animals from the group MS+SUC spending less time in the light compartment (Group MS+SUC – 3.0 ± 2.084% and Group C+SUC – 23.8 ± 4.889%, t = 3.91, df = 8, *p *< .05), showing longer latency periods (Group MS+SUC – 205.5s ± 34.50 and Group C+SUC – 43.0s ± 25.39, t = 3.89, df = 12, *p *< .05), and transitioning less often between the compartments (Group MS+SUC – 4.062 ± 1.451 and Group C+SUC – 10.50 ± 1.637, t = 2.68, df = 14, *p *< .05). On the other hand, no difference was observed between the groups MS+EtOH and C+EtOH (time spent in the light compartment: Group MS+EtOH – 5.481 ± 1.621% and Group C+EtOH – 13.41 ± 4.172%, t = 1.13, df = 19, *p *> .05; latency: Group MS+EtOH – 52.50s ± 17.50 and Group C+EtOH – 55.33s ± 25.34, t = 0.09, df = 19, *p *> .05; and number of compartment transitions: Group MS+EtOH – 4.33 ± 1.027 and Group C+EtOH – 7.88 ± 2.170, t = 0.52, df = 33, *p *> .05) (Figure 4d–f). Furthermore, two‐way analysis showed that C group and its interaction with sucrose consumption affected the time of permanence in the light compartment (*F*_1,21_ = 14.82, *p *< .05 and (*F*_1,21_ = 5.11, *p *< .05, respectively) and the latency time (*F*_1,31_ = 11.00, *p *< .05). However, the number of transitions between the two compartments was affected only by sucrose consumption (*F*_1,30_ =8.27, *p *< .05).

### 3.4 Effects of maternal separation and ethanol consumption in Blood Corticosterone Levels

**Figure 5:**
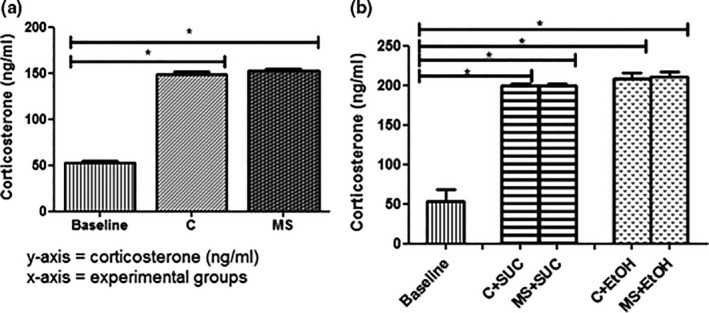
Plasma corticosterone concentration (ng/ml). Baseline values and concentrations for each group at the end of experiment 1 (T1) (a) and experiment 2 (T2) (b). Experimental design is described in Figure 1. All experimental groups were composed of seven animals (*n *= 7). Data are expressed as mean and standard error. One‐way ANOVA and Bonferroni's post hoc test correction were used to determine statistically significant differences between the baseline values and the groups C and MS in (a) and C+SUC, MS+SUC, C+EtOH and MS+EtOH in (b). *p < .05 baseline versus C and baseline versus MS in (a); and *p < .05 baseline versus C+SUC, baseline versus MS+SUC, baseline versus C+EtOH, and baseline versus MS+EtOH in (b)

Blood corticosterone concentration was measured at PND‐4 (baseline), PND‐22 (end of T1) and PND‐45 (end of T2) to determine the effects of maternal separation and ethanol or sucrose consumption in corticosterone levels (Figure 5). At the end of T1, groups MS (152.2 ± 2.112 ng/ml) and C (148.6 ± 2.776 ng/ml) showed increased corticosterone concentrations when compared with the baseline levels (52.46 ± 2.445 ng/ml, *F*_2,20_ = 451.9, *p *< .05) (Figure 5a). Similarly, animals from groups C+SUC (199.9 ± 1.899 ng/ml), MS+SUC (199.3 ± 2.631 ng/ml), C+EtOH (208.1 ± 7.382 ng/ml), and MS+EtOH (211.2 ± 5.555 ng/ml) presented higher levels of corticosterone in comparison with the baseline (53.59 ± 14.80 ng/ml, *F*_4,14_ = 98.53, *p *< .05) (Figure 5b). Interestingly, no differences were observed between the experimental groups, both at PND‐22 and PND45.

### 3.5 Effects of maternal separation in ethanol and sucrose consumption

**Figure 6:**
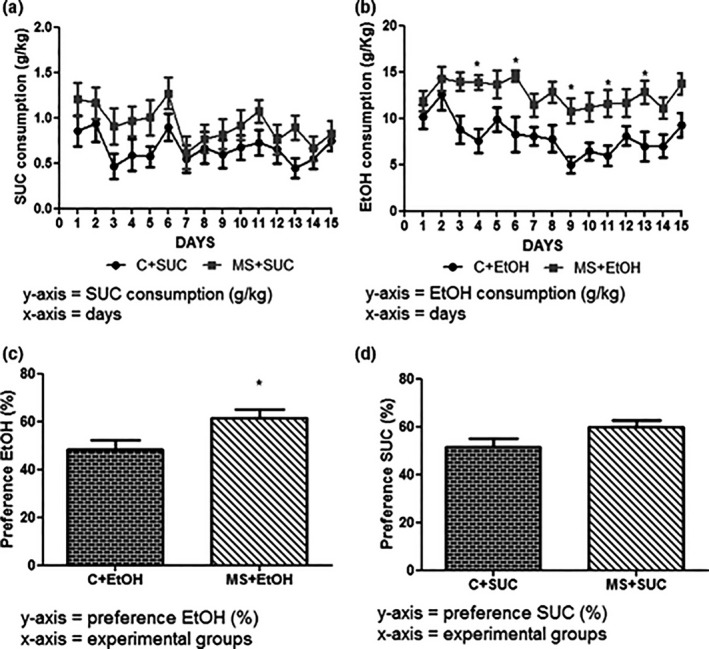
Consumption of and preference for Sucrose and Ethanol at the end of experiment 2 (T2). Consumption (g/Kg) of Sucrose (SUC) (a) and Ethanol (EtOH) (b). Preference (%) for EtOH versus water (c) or SUC versus water (d). Experimental design is described in Figure 1. All experimental groups were composed of seven animals (*n *= 7). Data are expressed as mean and standard error. Two‐way ANOVA and Bonferroni's post hoc test correction were used to determine statistically significant differences between the groups C+SUC and MS+SUC in (a) and between the groups C+EtOH and MS+EtOH in (b). Student's t‐test was used to determine statistically significant differences between the groups in C+SUC and MS+SUC in (c) and between the groups C+EtOH and MS+EtOH in (d). *p < .05 C+EtOH versus MS+EtOH

The ethanol or sucrose consumption was evaluated by daily quantifying the intake of these solutions during T2 with the aim of comparing the amount of ethanol ingested by the groups C and MS, and to verify if the animals that were separated from the mothers had preference for ethanol. Results are presented in grams per kilogram of body weight for each of the 15 days of the free‐choice model (Figure 6).

No differences were observed in sucrose intake between the groups C+SUC (0.662 ± 0.038 g/kg) and MS+SUC (0.923 ± 0.049 g/kg, *F*_14,22_ = 0.52, *p *> .05) (Figure 6a). However, animals in group MS+EtOH consumed more ethanol (12.63 ± 0.338 g/kg) than those in group C+EtOH (8.123 ± 0.485 g/kg, *F*_14,23_ = 2.53, *p *< .05) (Figure 6b).

The evaluation of the animals’ preference for sucrose/ethanol versus water corroborated these results. Preference was calculate using the formula: [total amount of ethanol or sucrose solution consumed/total amount of fluid) × 100] (Stevenson et al., 2015). Mice from group MS+EtOH showed preference for the ethanol solution (61.54 ± 3.704%), in contrast with mice from group C+EtOH, which showed no preference (48.11 ± 4.049%, t = 2.45, df = 23, *p *< .05) (Figure 6c). Animals from groups MS+SUC and C+SUC also did not show any preference for sucrose (Group MS+SUC – 59.91 ± 2.878% and Group C+SUC – 51.56 ± 3.440%, t = 1.86, df = 22, *p *> .05) (Figure 6d). In addition, ethanol preference was affected only by MS (*F*_1,45_ = 9.39, *p *< .05).

### 3.6 Effects of maternal separation and ethanol consumption in the expression of HPA axis, serotonergic, and reward system genes

We assessed transcript levels of genes directly associated with stress response at the end of T1 and T2 in hypothalamic and hippocampal tissues, as previously described (Firk & Markus, 2007; Greetfeld et al., 2009; Murgatroyd et al., 2009; Wu et al., 2014). Additionally, expression of reward system genes was evaluated in striatal tissue at the end of T2, as previously described (Jonsson, Ericson, & Söderpalm, 2014; Rimondini, Arlinde, Sommer, & Heilig, 2002). Seven animals were analyzed in each group (Groups C and MS in T1, and groups C+SUC, MS+SUC, C+EtOH, and MS+EtOH in T2).

**Figure 7:**
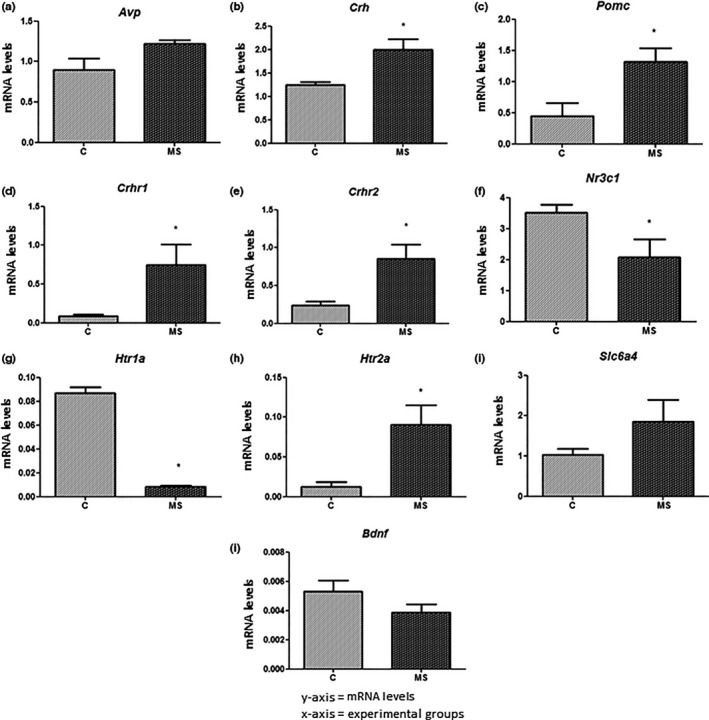
Relative mRNA levels in the hypothalamus (a, b and c) and hippocampus (d, e, f, g, h, i and j) at the end of experiment 1 (T1). (a) *Avp*: Arginine vasopressin. (b) *Crh*: corticotrophin‐release hormone. (c) P*omc:* pro‐opiomelanocortin‐alpha. (d) C*rhr1*: CRH receptor 1. (e) *Crhr2:* CRH receptor 2. (f) *Nr3c1:* glucocorticoid receptor. (g) *Htr1a*: serotonin receptor 5HT_1A_. (h) *Htr2a:* serotonin receptor 5HT_2A_. (i) *Slc6a4*: serotonin transporter. (j) *Bdnf:* Brain‐derived neurotrophic factor. Experimental design is described in Figure 1. All experimental groups were composed of seven animals (*n *= 7). Data are expressed as mean and standard error. The mRNA levels are expressed as arbitrary units. Student's t‐test was used to determine statistically significant differences between the groups C and MS. *p < .05 C versus MS

No significant difference in transcript levels of the gene Arginine‐vasopressin (*Avp*) was detected between the groups C and MS at the end of T1 (t = 1.92, df = 7, *p *> .05) (Figure 7a). Conversely, we observed an up‐regulation of the genes corticotrophin release hormone (*Crh*) and pro‐opiomelanocortin alpha (*Pomc*) in the group MS (*Crh*: 1.996 ± 0.227 and *Pomc*: 1.051 ± 0.225) in comparison with group C (*Crh:* 1.242 ± 0.068, t = 3.18, df = 10, *p *< .05 and *Pomc:* 0.814 ± 0.249, t = 2.65, df = 8, *p *< .05) (Figure 7b,c).

In hippocampal tissue samples, the receptors of corticotrophin‐releasing hormone *Crhr1* and *Crhr2* were up‐regulated in the MS group (0.7508 ± 0.2612 and 0.8534 ± 0.1876, respectively) compared with group C (0.0908 ± 0.0206, t = 2.78, df = 9, *p *< .05 and 0.2400 ± 0.0521, t = 3.50, df = 7, *p *< .05 respectively) (Figure 7d,e). On the other hand, the glucocorticoid receptor gene (*Nr3c1*) was down‐regulated in the MS group (2.074 ± 0.577) when compared with group C (3.503 ± 0.265, t = 2.24, df = 10, *p *< .05) (Figure 7f).

Regarding the serotonergic system, the serotonin receptor gene (*Hrt1a*) was down‐regulated in the group MS (0.0081 ± 0.0013) in comparison with group C (0.0869 ± 0.0047, t = 19.22, df = 8, *p *< .05) (Figure 7g). Contrarily, *Hrt2a* was up‐regulated in the first group (Group MS – 0.0905 ± 0.0250 and Group C – 0.0126 ± 0.0060, t = 3.30, df = 9, *p *< .05) (Figure 7h). The serotonin transporter (*Slc6a4)* and brain‐derived neurotrophic fator (*Bdnf)* genes showed no difference in transcript levels between the experimental groups (Figure 7i,j).

**Figure 8:**
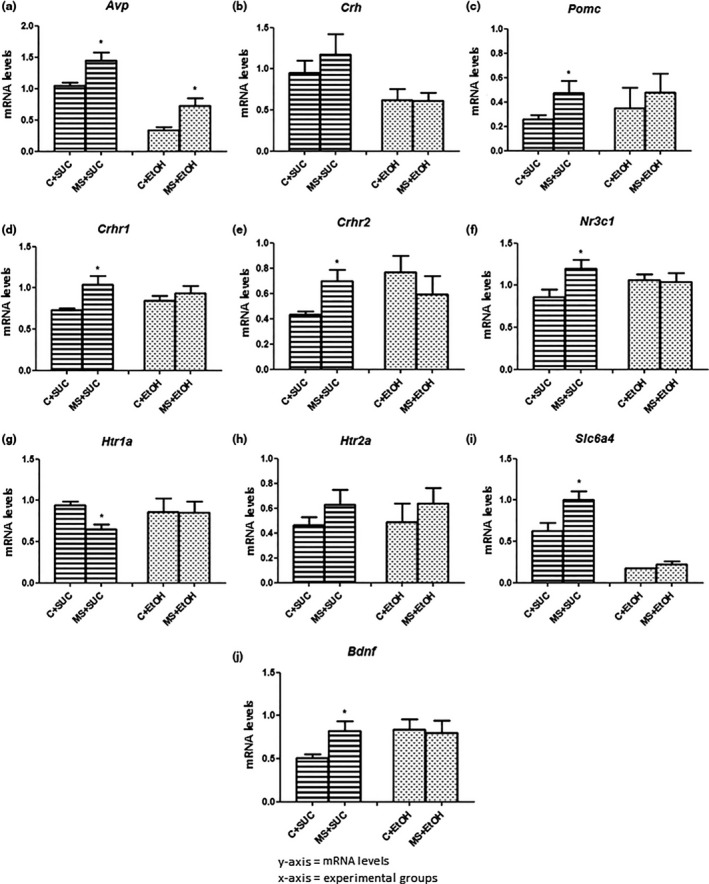
Relative mRNA levels in the hypothalamus (a, b and c) and hippocampus (d, e, f, g, h, i and j) at the end of experiment 2 (T2). (a) *Avp*: Arginine vasopressin. (b) *Crh*: corticotrophin‐release hormone. (c) P*omc:* pro‐opiomelanocortin‐alpha. (d) C*rhr1*: CRH receptor 1. (e) *Crhr2:* CRH receptor 2. (f) *Nr3c1:* glucocorticoid receptor. (g) *Htr1a*: serotonin receptor 5HT_1A_. (h) *Htr2a:* serotonin receptor 5HT_2A_. (i) *Slc6a4*: serotonin transporter. (j) *Bdnf:* Brain‐derived neurotrophic factor. Experimental design is described in Figure 1. All experimental groups were composed of seven animals (*n *= 7). Data are expressed as mean and standard error. The mRNA levels are expressed as arbitrary units. Student's t‐test was used to determine statistically significant differences between the groups C+SUC and MS+SUC and between the groups C+EtOH and MS+EtOH. *p < .05 C+SUC versus MS+SUC and C+EtOH versus MS+EtOH

All the above‐mentioned genes were also analyzed after the free‐choice model, at the end of T2. *Avp*, which was not differentially expressed between the groups in T1, was up‐regulated in the hypothalamus of mice from the groups MS+SUC (1.446 ± 0.1354) and MS+EtOH (0.7221 ± 0.1284) in comparison with the groups C+SUC (1.051 ± 0.0423, t = 2.28, df = 8, *p *< .05) and C+EtOH (0.3337 ± 0.0515, t = 2.59, df = 9, *p *< .05) respectively (Figure 8a). The transcript levels of *Avp* were affected by MS (*F*_1,17_ = 11.89, *p *< .05) and by ethanol consumption (*F*_1,17_ = 40.25, *p *< .05). In contrast, no differences were found in the expression of *Crh* between the groups (Figure 8b). However, the transcript levels of *Crh* in the hypothalamus were affected only by ethanol consumption (*F*_1,14_ = 8.15, *p *< .05). *Pomc* remained up‐regulated in mice from the group MS+SUC (0.4744 ± 0.0988) in comparison with those from group C+SUC (0.2562 ± 0.0349, t = 2.08, df = 10, *p *< .05) (Figure 8c). Similar to what we observed in T1, *Crhr1* and *Crhr2* were up‐regulated in the hippocampus of MS+SUC mice (1.037 ± 0.1070 and 0.7004 ± 0.0894, respectively) in comparison with C+SUC mice (0.7301 ± 0.0261, t = 2.78, df = 8, *p *< .05 and 0.4337 ± 0.0230, t = 2,88, df = 8, *p *< .05 respectively) (Figure 8d,e). The transcript level of *Crhr1* was affected only by MS (*F*_1,18_ = 6.29, *p *< .05). *Nr3c1*, which was down‐regulated in T1, was up‐regulated in the hippocampus of animals from the group MS+SUC (0.8617 ± 0.0879) when compared with those from group C+SUC (1.201 ± 0.1022, t = 2.51, df = 10, *p *< .05) (Figure 8f).

Analysis of the genes involved in the serotonergic system in samples of the hippocampus at the end of T2 showed down‐regulation of *Htr1a* in MS+SUC mice (0.6516 ± 0.0616) compared with C+SUC mice (0.9389 ± 0.0483, t = 3.55, df = 9, *p *< .05) (Figure 8g). And, in contrast with what we observed in T1, no differences were found in the mRNA levels of *Htr2a* between the groups in T2 (Figure 8h). On the other hand, *Slc6a4* and *Bdnf* (not differentially expressed at T1) were up‐regulated in the group MS+SUC (0.9988 ± 0.1089 and 0.8262 ± 0.1064, respectively) in comparison with group C+SUC (0.6311 ± 0.0942, t = 2.55, df = 10, *p *< .05 and 0.5082 ± 0.0473 t = 2.72, df = 10, *p *< .05 respectively) (Figure 8i,j). Furthermore, *Slc6a4* transcript levels were affected by MS (*F*_1,18_ = 6.11, *p *< .05) and by ethanol consumption (*F*_1,18_ = 54.66, *p *< .05).

**Figure 9:**
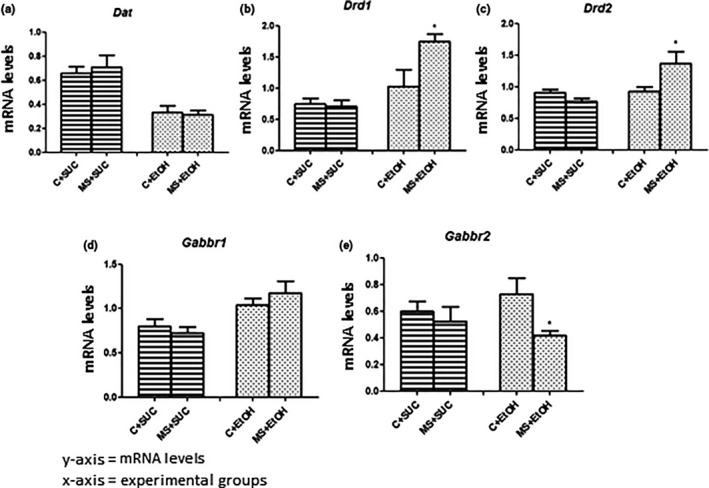
Relative mRNA levels in the striatum at the end of experiment 2 (T2). (a) *Dat:* dopamine transporter. (b) *Drd1:* dopamine receptor 1. (c) *Drd2:* dopamine receptor 2. (d) *Gabbr1:* GABAB receptor subunit 1. (e) *Gabbr2:* GABAB receptor subunit 2. Experimental design is described in Figure 1. All experimental groups were composed of seven animals (*n *= 7). Data are expressed as mean and standard error. The mRNA levels are expressed as arbitrary units. Student's t‐test was used to determine statistically significant differences between the groups C+SUC and MS+SUC and between the groups C+EtOH and MS+EtOH. *p < .05 C+EtOH versus MS+EtOH

At the end of T2, we also analyzed genes involved in the reward system in striatal tissue, in an attempt to identify molecular changes caused by ethanol consumption. No differences were found in the expression of the dopamine transporter gene (*Dat*) between the groups (Figure 9a). However, transcript levels of *Dat* were affected by the ethanol consumption (*F*_1, 18_ = 27.39, *p *< .05.) Dopamine receptors *Drd1* and *Drd2* were up‐regulated in the striatum of MS+EtOH mice (1.745 ± 0.1181 and 1.371 ± 0.1832, respectively) in comparison with C+EtOH mice (1.027 ± 0.2674, t = 2.65, df = 7, *p *< .05 and 0.9238 ± 0.0696, t = 2.49, df = 7, *p *< .05, respectively) (Figure 9b,c). *Drd1* and *Drd2* transcript levels were affected by ethanol consumption (*F*_1,17_ = 22.56, *p *< .05 and *F*_1,17_ = 12.71, *p *< .05, respectively) and by the interaction of MS and ethanol (*F*_1,17_ = 7.52, *p *< .05 and *F*_1,17_ = 11.11, *p *< .05 respectively). Furthermore, the transcript level of *Drd1* also was affected by MS (*F*_1,17_ = 5.98, *p *< .05). Expression of the gamma‐aminobutyric acid receptor (GABA_B_) subunit *Gabbr1* showed no difference among the groups (Figure 9d), while the subunit Gabbr2 was down‐regulated in MS+EtOH mice (0.4185 ± 0.0341) when compared with C+EtOH mice (0.7281 ± 0.1211, t = 2.67, df = 9, *p *< .05) (Figure 9e). The transcript levels of *Gabbr1* were affected only by ethanol consumption (*F*_1,18_ = 11.52, *p *< .05), while the expression of *Gabbr2* was affected only by MS (*F*_1,18_ = 4.79, *p *< .05).

## 4 DISCUSSION

Given the important role of postnatal stress as a predictor of ethanol consumption, we described here a mouse model that employed a maternal separation protocol (adapted from Nylander & Roman, 2013) followed by 15 days of free‐choice between water and ethanol solution (Crabbe et al., 2011). We showed that maternal separation can influence the expression of genes involved in stress response and increase pups’ vulnerability to ethanol consumption during adolescence. Furthermore, we demonstrated, for the first time, the effects of ethanol consumption on the stress response and on the regulation of the reward system genes.

We used both C57BL/6 males and females since no differences were observed between the sexes in all the experiments performed (Figures S1, S2, S3, S4 and S5). Therefore, we established that maternal separation equally alters the anxiety/stress response in male and female pups. Different anxiety‐related behavior patterns between C57BL/6 adult males and females have been previously reported in females in diestrous phase (75 days old) (Romeo et al., 2003). In our study, however, the age of the females did not exceed 45 days, and they were under low hormonal fluctuations, as the sexual maturity of these mice occurs between PND‐50 and PND‐60 (Carrier, Wang, Sun, & Lu, 2015). Furthermore, the majority of the studies involving MS have partially addressed sex differences in its outcome. Although few studies have shown that MS resulted in faster behavioral sensitization to ethanol in female mice (Kawakami et al., 2007; Nylander & Roman, 2013), other studies compared male and female rodents and reported no sex differences in the ethanol consumption (Lancaster, Brown, Coker, Elliott, & Wren, 1996; Nylander & Roman, 2013; Weinberg, 1987), which is in accordance with our results.

Our maternal separation protocol affected weight gain in mice from the group MS in comparison with group C. This was expected as mice depend on their mothers for feeding on the first PNDs (Kawakami et al., 2007). During T2, however, no differences were observed in weight gain between the experimental groups, which was also expected given that maternal separation was interrupted in this experiment.

Evaluation of maternal behavior in T1 revealed stronger care taking behavior of the mothers towards the MS pups after separation compared with the rest of their offspring, which were kept with them. Previous findings have shown that increased maternal care after maternal separation in C57BL/6 mice can reduce anxiety and stress, as indicated by lower anxiety and unaltered corticosterone levels in these mice (Own & Patel, 2013). These results support our data as we observed no differences in corticosterone levels and in the light/dark box behavioral test between the groups MS and C at the end of T1.

It is important to notice that we observed a doubling in nursing behavior in group C in postseparation treatment, which was similar in magnitude to the change seen in the breastfeeding behavior in the MS group. This evidence of stress‐like effects in the C group could explain the fact that neither the HPA axis output nor behavioral measures reflected a stress effect in the MS paradigm. However, this control condition is the most frequently used (85.41%) in studies of MS (Tractenberg et al., 2016). Early handling procedures reflect animals’ natural conditions (Tractenberg et al., 2016) and provide the preferential control group for studies of prolonged MS (Nylander & Roman, 2013). Any early manipulation procedure (e.g. brief manipulations by experimenters to mark pups, record weight, clean or change cages) prepares the pups for future manipulations so that control pups tend to be less responsive to them.

Additionally, the choice of keeping the control group in the same cage as the MS group is thought to mimic conditions in the wild (Tractenberg et al., 2016). Another concern in choosing the control group is the impact on the development of infant brain behavior during litter handling or cross‐fostering, so that different control conditions may produce different brain behavior data (Barbazanges et al., 1996; Maccari et al., 1995). In this context, control groups in which animals are separated from the MS group are limiting, since they may influence brain behavior, and there is no control for the effects of handling per se and may therefore interact with the effects of MS (Lehmann & Feldon, 2000). Based on this evidence, the inbred control chosen has attempted to undermine litter / genetic disorders since it has the advantage of evaluating the effects among related animals that are genetically similar (Lehmann & Feldon, 2000; Zimmerberg & Shartrand, 1992). Thus, differences in neuroendocrinology / behavior may be more easily detected because of the reduction in interindividual variability (Lehmann & Feldon, 2000).

Expression of the main genes involved in the HPA axis and the serotonergic system was altered at the end of T1, with MS mice showing (i) up‐regulation of *Crh* and *Pomc* in the hypothalamus; (ii) up‐regulation of *Crhr1* and *Crhr2* in the hippocampus; and (iii) down‐regulation of *Nr3c1* in the hippocampus in comparison with the control group (C). These results are in agreement with previous studies that have described increased levels of *Crhr1* and *Crhr2* in the hippocampus after maternal separation (Lee, Schmidt, Tilders, & Rivier, 2001; Plotsky et al., 2005; Wang et al., 2014), and have shown that reduced *Nr3c1* mRNA levels have a deleterious impact in the negative feedback regulation of glucocorticoids, leading to higher concentrations of CRH and POMC in the hypothalamus and of ACTH in the pituitary (Frodl & O'Keane, 2013; McGowan et al., 2009).

We did not observe any differences in the expression of *Avp* after T1, similar to what has been observed in other early life stress studies (Oreland, Gustafsson‐Ericson, & Nylander, 2010; Todkar et al., 2015). Nevertheless, higher expression levels of *Avp* and *Nr3c1* after maternal separation have been previously reported (Murgatroyd et al., 2009). The divergence with our data, however, might reflect methodological differences, since the mentioned study used a protocol with three hours of maternal separation between days PND‐1 and PND‐10, while we separated the animals for six hours daily between PND‐5 and PND‐21.

Despite the importance of the regulation of serotonergic system genes and their correlation with the HPA axis, few studies have established the relationship between these two systems (Gartside and Cowen 1990; Own et al., 2013). Here we showed that, after maternal separation, *Htr1a* was down‐regulated and *Htr2a* was up‐regulated in the hippocampus of mice from the MS group compared with the control group (C). Our data corroborates the findings of Firk and Markus (2007), which showed a decrease in *Htr1a* in the hippocampus following stress situations, and of Maple, Zhao, Elizalde, McBride, and Gallitano (2015), which demonstrated that acute stress increases *Htr2a* in the same structure. Additionally, these changes in serotonergic transmission are also responsible for the excessive activation of the HPA axis (Herman et al., 2005). Our results, in combination with these data, suggest that the serotonergic system plays an important role in the regulation of stress and could induce changes in the HPA axis, possibly mediated by 5‐HT1A/2A receptors.

Early life stress events are associated with vulnerability to excessive voluntary ethanol consumption (Nylander & Roman, 2013). In our model, animals subjected to maternal separation (Group MS+EtOH) consumed more ethanol and presented higher preference for ethanol solution during adolescence (PND‐30 to PND‐45) when compared with their respective controls (Group C+EtOH). No differences were observed in the consumption and preference for sucrose between the groups MS+SUC and C+SUC. This suggests that animals from the MS group consumed ethanol for stress relieving purposes; and can probably be explained by the normalization of the HPA axis and serotonergic system genes (differentially expressed in group MS after T1), following exposure to ethanol in T2 ‐ i. e., the consumption of ethanol was able to normalize the expression of stress‐related genes, thus, alleviating stress. Although previous studies have reported that postnatal stress can alter HPA axis activity and increase the risk of ethanol consumption, their analysis were restricted to the corticosterone levels, and did not identify the genes or the molecular mechanisms underlying the role of the ethanol in alleviating stress (Bendre et al., 2015; Enoch, 2011; Mohn et al., 2011; Todkar et al., 2015).

Here we analyzed, for the first time, the transcript levels of stress response genes following the free‐choice model (T2), and determined that mRNA levels of the genes *Crh*,* Pomc*,* Crhr1*,* Crhr2*,* Nr3c1 Htr1a*,* Htr2a*,* Slc6a4,* and *Bdnf* were similar between the groups MS+EtOH and C+EtOH, thus, indicating that ethanol intake was able to normalize the expression of stress‐related genes that were differentially regulated as a consequence of maternal separation (at the end of T1).

Considering that ethanol promotes its reward effects and alleviates stress via the activation of the mesolimbic dopaminergic pathway in the reward system (Koob & Volkow, 2010; Nestler, 2005; Spanagel, 2009), we also evaluated the main genes involved in this process. We observed no difference in the expression of *Dat* and *Gabbr1*, but *Drd1* and *Drd2* were up‐regulated and the subunit *Gabbr2* was down regulated in the striatum of MS+EtOH mice in comparison with C+EtOH mice. Interestingly, overexpression of *Drd2* has been previously associated with a significant decrease i*n *ethanol consumption in rats (Thanos et al., 2004), which suggests that ethanol effects could be mediated by *Drd2*. Our results are in accordance with previous studies linking excessive ethanol consumption to an increase in *Drd1* and *Drd2* expression and to an imbalance between the subunits of GABA_B_ receptors (Delis et al., 2015; Ribeiro et al., 2012). This, together with the differential regulation of the reward system in these animals, confirms our hypothesis that mice from the MS group consume ethanol to alleviate stress.

In accordance with this hypothesis, the light/dark box behavioral test at the end of T2 did not reveal behavioral differences between the groups MS+EtOH and C+EtOH, and no differences were found in the corticosterone levels between these groups. The lack of a difference in corticosterone levels between water‐ and alcohol‐drinking individuals has been previously reported in rats that suffered maternal separation, and, again, suggests that they consumed ethanol for attenuation of stress (Bendre et al., 2015). Unlike the animals that received ethanol, mice in the MS+SUC group presented anxiety/stress‐related behavioral changes in comparison with group C+SUC, thus corroborating the conclusion that the ethanol was able to mask the effects of stress.When evaluating the genes of the HPA axis and the serotonergic system in animals that received sucrose, we observed that *Pomc* remained up‐regulated in the hypothalamus and *Crhr1* and *Crhr2* remained up‐regulated in the hippocampus of MS+SUC mice in comparison with C+SUC mice, similar to what we observed for the MS and C mice in T1. However, *Crh*, which was up‐regulated in MS mice after T1, was not differentially expressed among the different groups after T2. *Avp*, on the other hand, was up‐regulated in the hypothalamus of MS+SUC and MS+EtOH mice in comparison with C+SUC and C+EtOH mice, respectively ‐ despite being expressed at similar levels in the groups MS and C after T1. These results are in accordance with previous studies showing that *Crh* expression progressive and significantly decreases while the levels of *Avp* significantly increase with age, in the hypothalamus of rodents (Cizza et al., 1994; Keck et al., 2000). This suggests an age dependent mechanism of adaptation to stressful conditions, in which the importance of hypothalamus‐derived AVP for the regulation of the HPA axis increases with age, while the importance of CRH decreases (Goncharova, 2014). These observations might also explain the up‐regulation of *Pomc*,* Crhr1* and *Crhr2* as a response to maternal separation. The *Nr3c1* gene, which was down‐regulated in MS mice after T1, was up‐regulated in the hippocampus of MS+SUC mice compared with C+SUC mice after T2. Similar results were found in previous studies that reported an increase in *Nr3c1* expression with age as an adaptation to stress (Harris & Saltzman, 2013).

In the hippocampus, *Htr1a* remained down‐regulated in the group MS+SUC compared with group C+SUC. However, *Htr2a,* which was up‐regulated in the MS group after T1, was similarly expressed in the groups MS+SUC and C+SUC at the end of T2. The genes *Bdnf* and *Slc6a4*, not differentially expressed after T1, were up‐regulated in MS+SUC mice compared with C+SUC mice at the end of T2. Evidence suggests that the expression levels of 5HT receptors in the limbic structures gradually decrease from infancy to adolescence (Lidow, Goldman‐Rakic, & Rakic, 1991; Slotkin, Cousins, Tate, & Seidler, 2005). These and our results are in agreement with a study showing that the higher levels of 5HTT found in young rhesus monkeys in comparison with older ones was linked to age (Embree et al., 2013). In contrast, other investigators have shown that maternal separation can lead to decreased BDNF in the hippocampus of rats (García‐Gutiérrez et al., 2015; Lippmann, Bress, Nemeroff, Plotsky, & Monteggia, 2007). The divergence with our data might be explained by the different species or the maternal separation protocol used. Further analyzes, beyond the scope of this study, are needed to clarify the nature of the molecular alterations observed in adolescent MS mice and their effects on ethanol consumption, as well as the changes in the reward system.

We adopted here a model that we considered appropriate for the specificities of our investigation. It is important to highlight, however, that future studies in this area should consider the systematic overview of all the relevant material regarding maternal separation and voluntary ethanol consumption to explore the methodological differences that might underlie inconsistencies in the preclinical investigations of the effects of postnatal stress and/or ethanol consumption (Tractenberg et al., 2016). In summary, we showed here that the postnatal stress of maternal separation increased pups’ vulnerability to ethanol consumption in adolescence, as indicated by the observations that mice in the MS group presented: (i) changes in anxiety/stress‐related behavior; (ii) increased EtOH consumption; and (iii) changes in the expression levels of key genes of the HPA axis, and the serotonergic and reward systems after maternal separation and the free‐choice protocol. Additionally, our data suggest that ethanol consumption relieves stress symptoms by reducing the activity of the HPA axis and the serotonergic system at the end of T2 (Group MS+EtOH), these same changes were not found independent of the time (T1 and T2) in animals that did not have access to ethanol highlighting the importance of assessing the genes involved in stress response in the postnatal (before exposure to the ethanol) and adolescence period (after exposure to ethanol). Our findings may help in future studies designed to clarify the molecular interactions of this phenotype, may help to further understand the pathogenic processes underlying psychiatric disorders and ethanol consumption, and may also contribute to the development of therapeutic strategies for these conditions.

## CONFLICT OF INTEREST

The authors declare that there are no conflicts of interest.
