# Supplementary figures and images for: Maternal separation affects expression of stress response genes and increases vulnerability to ethanol consumption
Source: Brain Behav. 2017 Nov 30;8(1):e00841. doi: 10.1002/brb3.841 (PMC5853632; doi:10.1002/brb3.841)

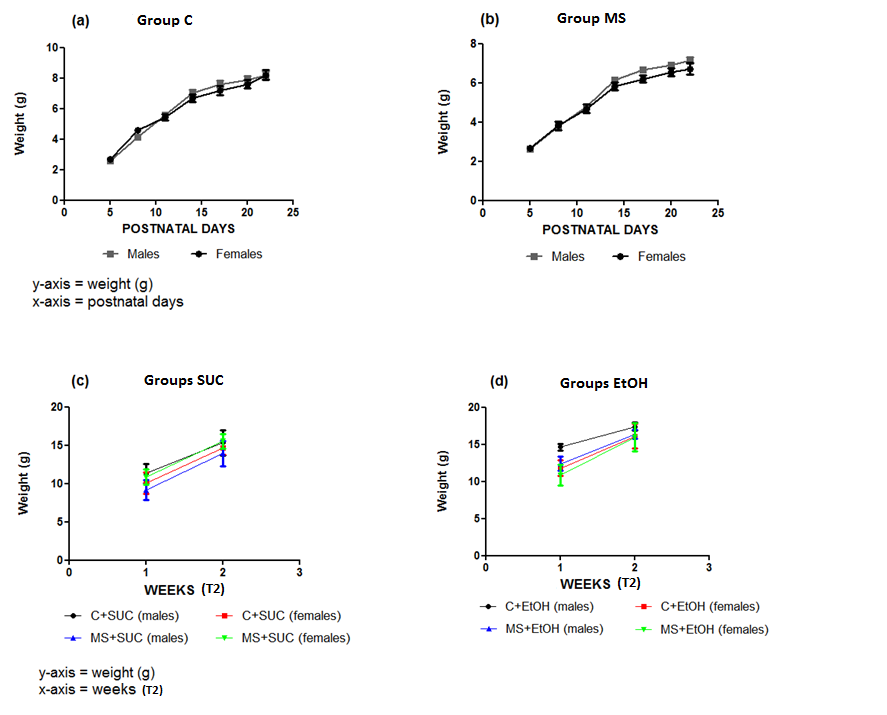

Supplement: Supplementary file 1 [file BRB3-8-e00841-s001.tif]

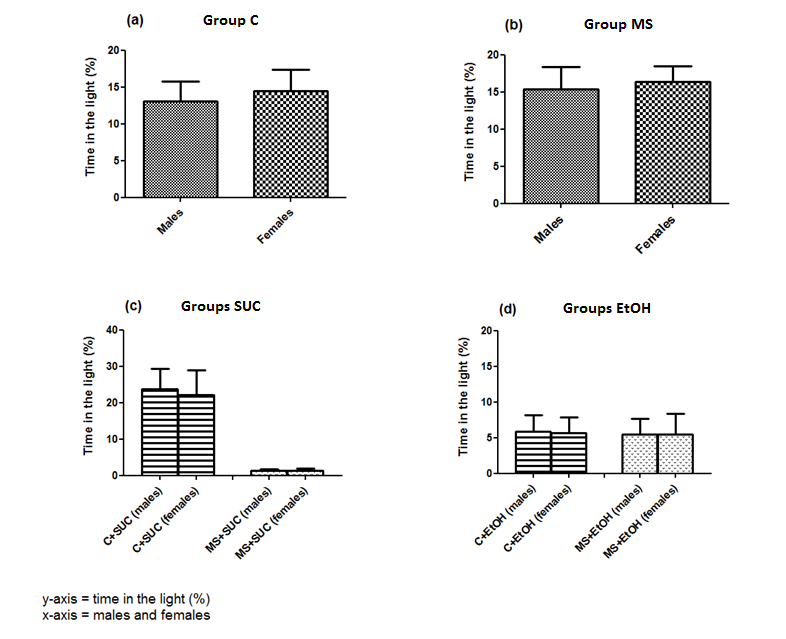

Supplement: Supplementary file 2 [file BRB3-8-e00841-s002.tif]

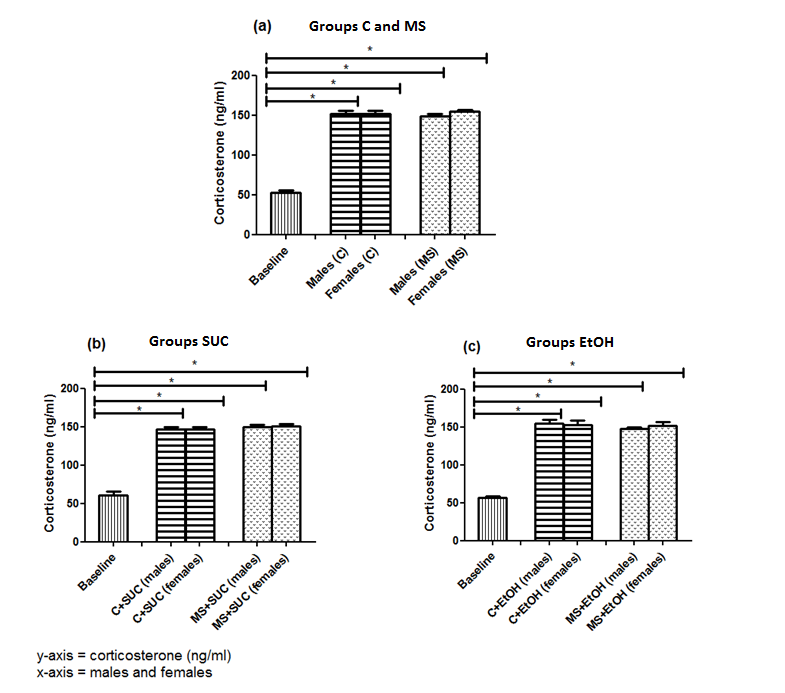

Supplement: Supplementary file 3 [file BRB3-8-e00841-s003.tif]

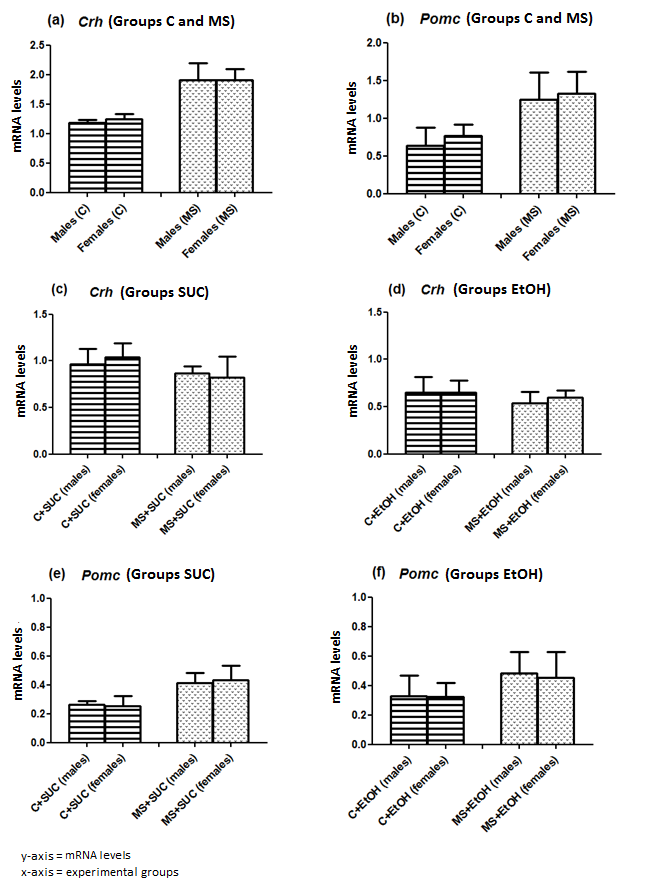

Supplement: Supplementary file 4 [file BRB3-8-e00841-s004.tif]

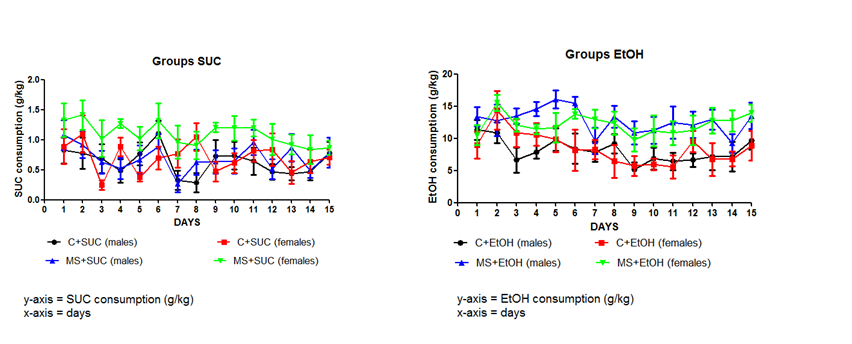

Supplement: Supplementary file 5 [file BRB3-8-e00841-s005.tif]
